# Supplementary figures and images for: Oceanographic moorings as year-round laboratories for investigating growth performance and settlement dynamics in the Antarctic scallop Adamussium colbecki (E. A. Smith, 1902)
Source: PeerJ. 2019 Mar 21;7:e6373. doi: 10.7717/peerj.6373 (PMC6431546; doi:10.7717/peerj.6373)

# Von Bertalanffy growth functions for *Adamussium colbecki*

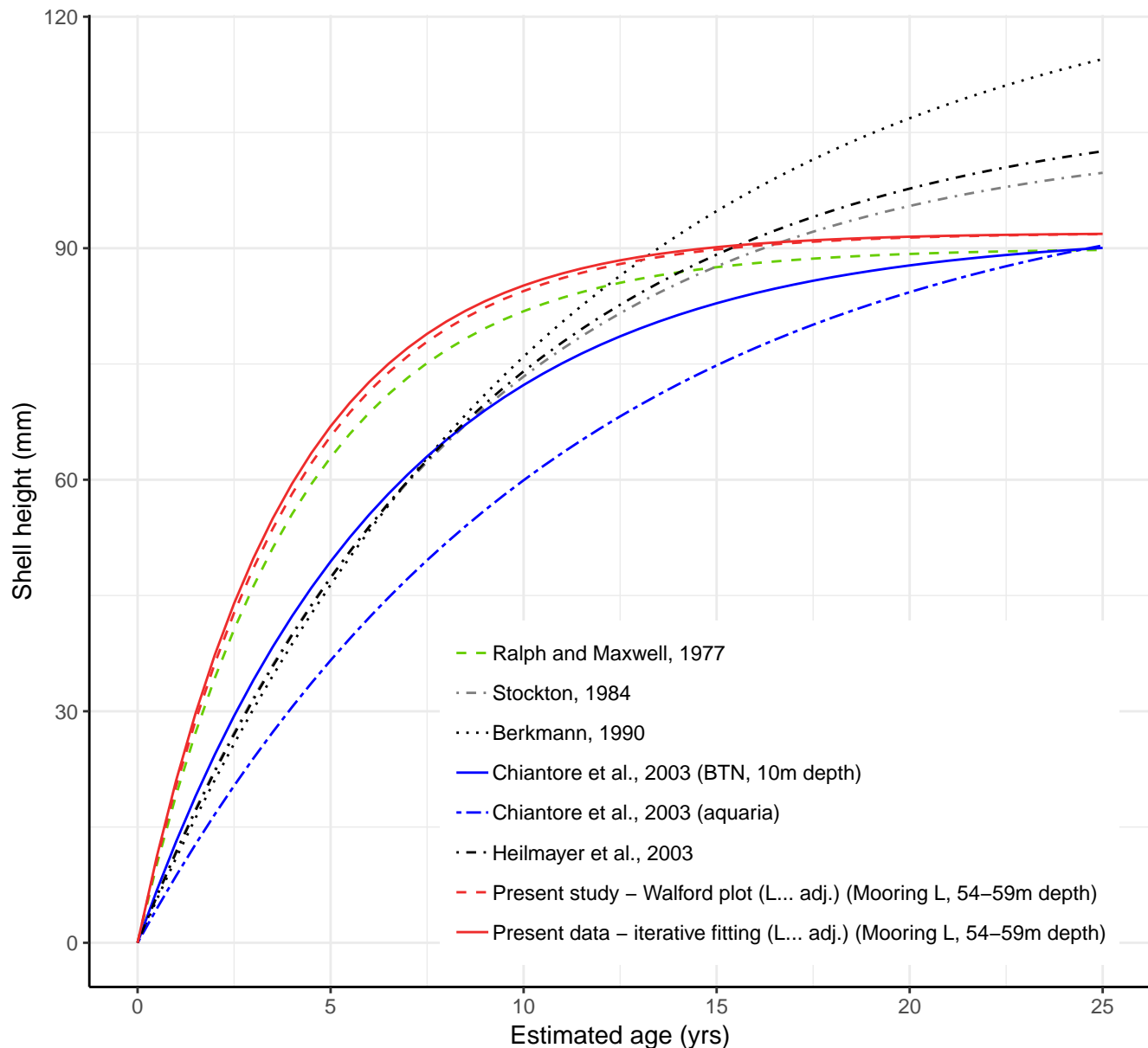

Supplement: Supplemental Information 1 — von Bertalanffy growth functions from present and literature data. In this case L∞was set to 92 mm following Chiantore, Cattaneo-Vietti & Heilmayer (2003); see text for details. [file peerj-07-6373-s001.pdf]

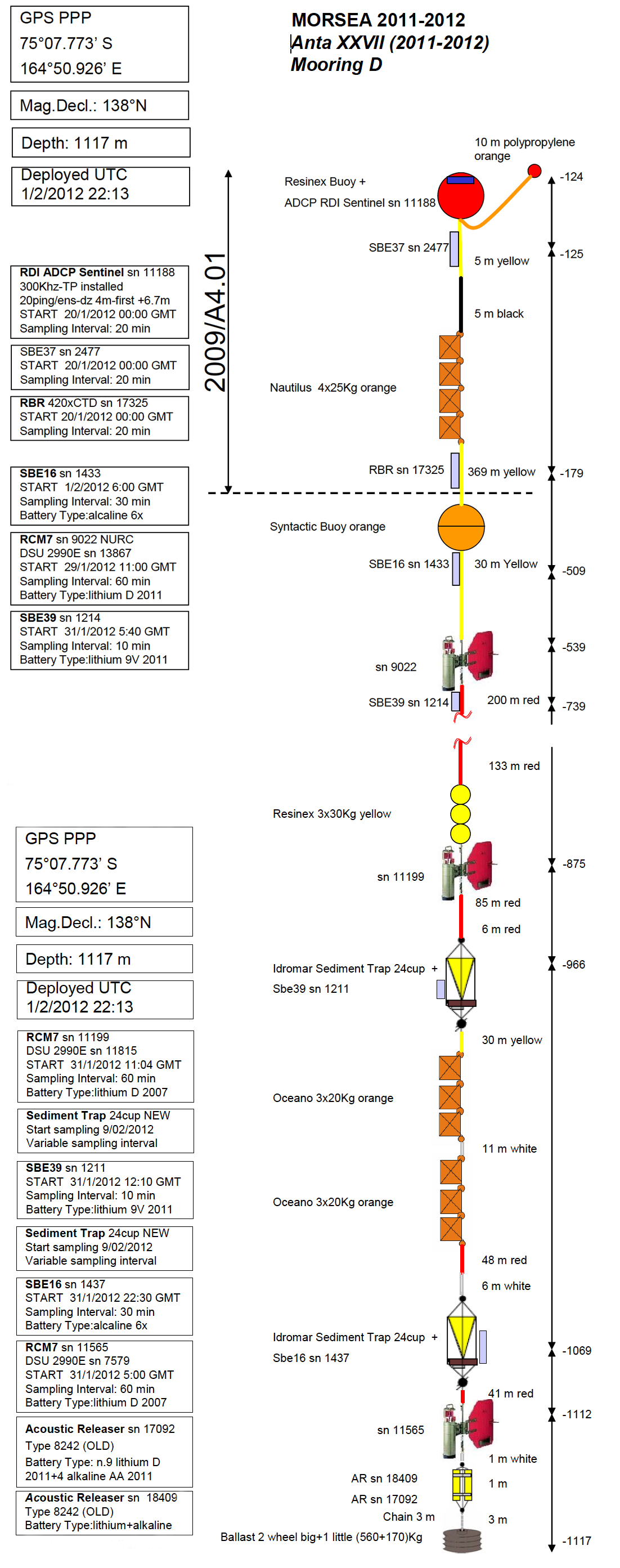

Supplement: Supplemental Information 2 — Mooring “D” was placed at a depth of 1086 m in the polynya facing the Drygalski Ice Tongue (75°07.773’S; 164°50.926’E), which is one of the most important areas for the production of High Salinity Shelf Waters (HSSW) in the Ross Sea. [file peerj-07-6373-s002.jpg]

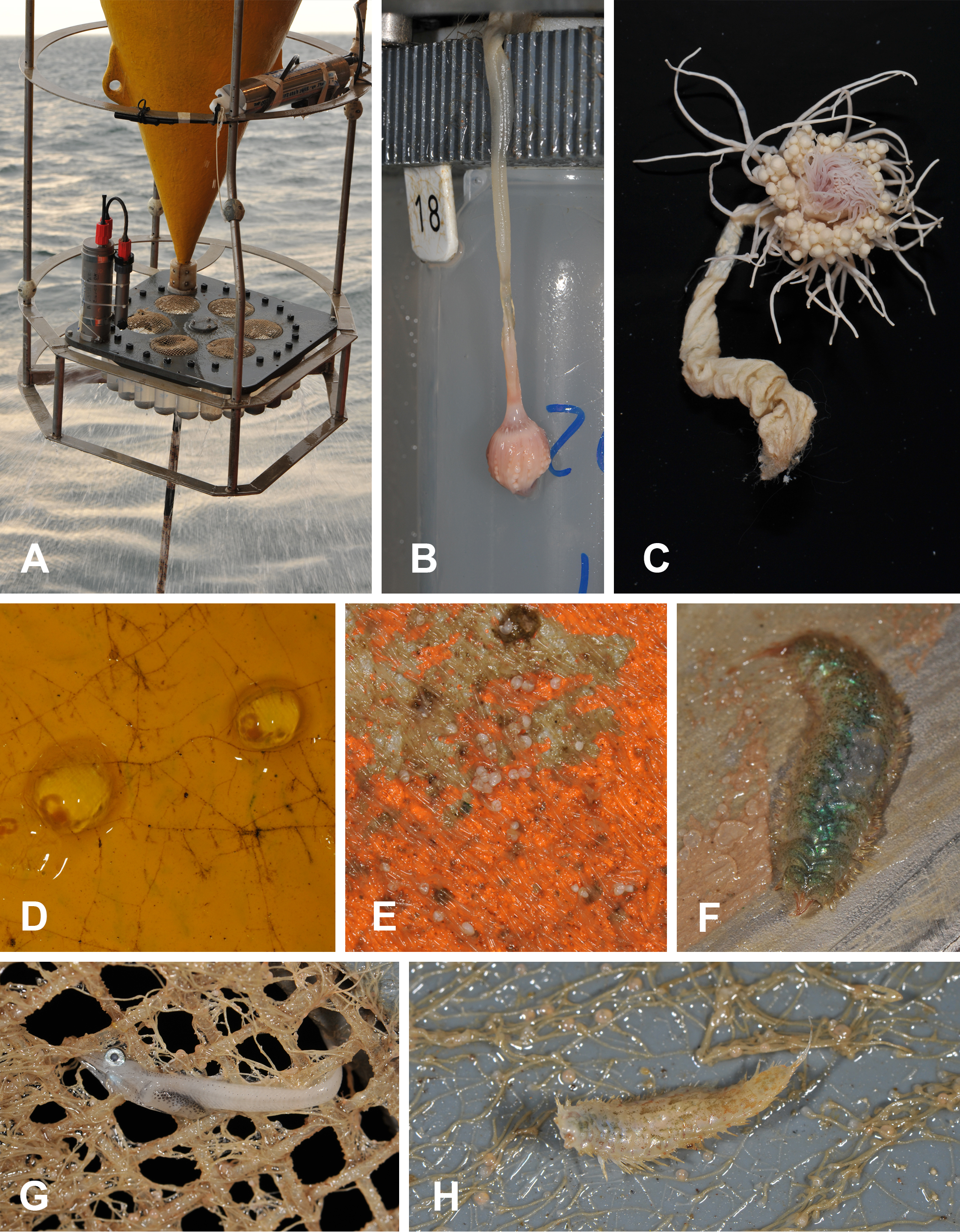

Supplement: Supplemental Information 3 — (A) The sediment trap structure. (B) The hydroid Monocaulus sp. (MNA 5469) attached to the sediment bottles’ carousel. (C) The same specimen photographed in the lab immediately after collection. (D) Unidentified solitary ascidians. The specimens in the picture have not been sampled, but the same species was been collected on another occasions and is available in the MNA collections (MNA 10546). (E) Network of hydroids and empty shells of Limacina rangii (d’Orbigny, 1835). (F) The polynoid polychaete Harmothoe fuligineum(Baird, 1865) (MNA 3403). (G) Larva of an unidentified ‘ice-fish.’ (H) Another polynoid polychaete, Harmothoe sp. (MNA 3415). [file peerj-07-6373-s003.jpg]
